# Factors associated with self-rated health in older people living in institutions

**DOI:** 10.1186/1471-2318-8-5

**Published:** 2008-02-27

**Authors:** Javier Damián, Roberto Pastor-Barriuso, Emiliana Valderrama-Gama

**Affiliations:** 1 National Centre for Epidemiology, "Carlos III" Institute of Health, Madrid, Spain; 2 "Campo de la Paloma" Primary Care Center, IMSALUD, Madrid, Spain

## Abstract

**Background:**

Although self-rated health has been extensively studied in community older people, its determinants have seldom been investigated in institutional settings. We carried out a cross-sectional study to describe the physical, mental, and social factors associated with self-rated health in nursing homes and other geriatric facilities.

**Methods:**

A representative sample of 800 subjects 65 years of age and older living in 19 public and 30 private institutions of Madrid was randomly selected through stratified cluster sampling. Residents, caregivers, physicians, and nurses were interviewed by trained geriatricians using standardized instruments to assess self-rated health, chronic illnesses, functional capacity, cognitive status, depressive symptoms, vision and hearing problems, and social support.

**Results:**

Of the 669 interviewed residents (response rate 84%), 55% rated their health as good or very good. There was no association with sex or age. Residents in private facilities and those who completed primary education had significantly better health perception. The adjusted odds ratio (95% confidence interval) for worse health perception was 1.18 (1.07–1.28) for each additional chronic condition, 2.37 (1.38–4.06) when comparing residents with moderate dependency to those functionally independent, and 10.45 (5.84–18.68) when comparing residents with moderate/severe depressive symptoms to those without symptoms. Visual problems were also associated with worse health perception. Similar results were obtained in subgroup analyses, except for inconsistencies in cognitively impaired individuals.

**Conclusion:**

Chronic conditions, functional status, depressive symptoms and socioeconomic factors were the main determinants of perceived health among Spanish institutionalized elderly persons. Doubts remain about the proper assessment of subjective health in residents with altered cognition.

## Background

Self-rated health is a complex variable that captures multiple dimensions of the relation between physical health and other personal and social characteristics. It is very consistent its capacity to predict mortality [1] and functional loss [2,3], independently of objective health, psychosocial, and demographic variables. It has also been strongly associated with successful aging [4] and evidence of biologic roots has been recently shown [5,6]. Self-rated health is very easy to obtain through a single-item question and, consequently, it is often included in health surveys and as an outcome in many studies, resulting in a large body of research. However, few studies have focused on the determinants of self-rated health in institutionalized older people. One early study found a positive association between subjective and objective health in community older persons, but not in those living in institutions [7]. Another study confirmed the association of self-rated health with mortality in institutionalized Chinese elderly, but did not offer relevant information on its determinants [8].

The singular physical, psychosocial, and environmental characteristics of institutionalized older people justify the study of determinants of subjective health in these populations. The present study aims at identifying the principal determinants of self-rated health among a representative sample of older people living in institutions in Madrid, Spain. We also aimed at exploring potential effect modifications by sex, type of facility, and cognitive status, since differences in socioeconomic status and delivery of care among residents of public and private facilities, as well as the ability of cognitively impaired subjects to make self evaluations, might condition the effect of other variables on subjective health.

## Methods

### Population and selection of participants

Between June 1998 and June 1999, we conducted a survey based on a probabilistic sample of residents 65 years of age and older of public and private nursing homes in the city of Madrid and a surrounding area of 35 km. Study participants were selected through stratified cluster sampling. Sampling was stratified by the funding of the institution: one stratum included 22 public and 25 concerted (privately owned but publicly funded) nursing homes, and the other stratum included 139 private institutions. In the first sampling stage, we sampled 19 public/concerted and 30 private institutions with probability proportional to its size. In the second stage, we randomly sampled 10 men and 10 women within each selected public/concerted facility (6 large facilities comprised two clusters each, thus selecting 20 men and 20 women), yielding a sample of 500 residents from public/concerted nursing homes. Similarly, we sampled 5 men and 5 women from each selected private nursing home, totalling 300 residents in this stratum. As a result of this selection procedure, residents of public institutions and men were over-sampled in order to improve precision in these subgroups.

The institutional review board of the "Carlos III" Health Institute approved the study. Informed consent was obtained from all subjects (or next of kin) studied.

### Data collection

Trained geriatricians or residents in geriatrics used structured questionnaires to collect information by interviewing the residents, their main caregivers, and the physician (or nurse).

#### Socio-demographic variables

Each resident's sex, age, and educational level (less than primary, primary -8 years-, and secondary -12 years- or more) were obtained by interview.

#### Self-rated health

Residents were asked about their health through the question: "In general terms, how would you describe your health: very good, good, fair, poor, or very poor?" No health-related questions that could influence the response were made before asking people to rate their health.

#### Chronic conditions

Physicians (or nurses in 8% of cases) were asked whether the residents suffered from any of the following conditions: arthritis (including severe osteoarthritis), obstructive pulmonary disease (emphysema, asthma, or other chronic obstructive pulmonary disorder), diabetes, hypertension, anaemia, ischemic heart disease, congestive heart failure, peripheral arterial disease, arrhythmias, stroke, depression, Parkinson's disease, Alzheimer's disease, other dementias, epilepsy, and cancer.

#### Functional status

We used the Barthel index as modified by Shah et al [9]. Subjects (55%) or their main caregivers (if assigned, 45%) were asked as to the degree of dependency in performing the following basic activities of daily living: eating, going to the toilet, personal hygiene, bathing/showering, dressing/undressing, transferring, walking, use of stairs, and urinary/faecal continence. Residents were classified into the following categories: independent (100 points), mild dependency (91–99 points), moderate dependency (21–90 points), and severe dependency (0–20 points).

#### Cognitive status

Residents were subject to the Pfeiffer's Short Portable Mental Status Questionnaire [10] (SPMSQ, range 0–10 errors) with some modifications to adapt to the institutional setting. We also used the Minimum Data Set Cognition Scale (MDS-COGS) [11,12], which obtains an assessment from the main caregiver of the resident's cognitive status based on selected Minimum Data Set questions (0–10 point scale from intact to very severe impairment). With both scales we created a two-category variable: impaired cognition comprised persons with more than 3 education-adjusted errors in SPMSQ or more than 2 points in the MDS-COGS, while the rest formed the normal cognition group.

#### Depressive symptoms

Residents were asked to respond to a 10-item version of the Geriatric Depression Scale [13], with 0–3, 4–7, and 8–10 point ranges indicating normal, mild, and moderate/severe depressive symptoms, respectively [14].

#### Vision and hearing

These conditions were assessed by means of the two corresponding Minimum Data Set four-category questions [15], and then dichotomized as good/mild versus moderate/severe impairment. Residents or caregivers were asked to assess the residents' ability to see in adequate light and with glasses, if used; as well as their hearing status even with hearing appliance, if used.

#### Social interaction

These aspects were appraised with three questions, further dichotomized. One inquired on the degree of relationships and participation in activities in the institution (four categories collapsed into frequent/normal versus rare/nothing). Another asked for the frequency of outside contacts with family or friends (five categories collapsed into daily/weekly/monthly versus lower than monthly/nothing). The third one questioned on whether the resident had a family member in the facility.

### Analysis

We used ordered logistic regression models to obtain the odds of reporting worse health perception (five category response variable) as a function of multiple explanatory variables. The main model included sex, age (65–84 or ≥ 85 years), education (less than primary, primary, or secondary or more), type of facility (public/concerted or private), number of chronic conditions, and functional status (independent, mild, moderate, or severe dependency) as explanatory variables. We assessed the association of cognitive status, depressive symptoms, vision and hearing problems, and social interactions (internal, external contacts, and relatives in facility), adjusted for the above main model covariates. Finally, to evaluate potential effect modifications, additional models were fitted separately for each sex, type of facility, and cognition category group. Interaction tests were performed by contrasting product terms in models adjusted for main covariates.

Due to the complex sampling and the different selection probabilities of study participants, all analyses were weighted to the underlying population distribution and accounted for the effect of stratification and clustering on point and interval estimates. Analyses were run using Stata 8.1 statistical software [16].

## Results

We obtained information on self-rated health in 669 of the 800 residents in the initial sample (overall response rate 84%). In our population (weighted and design-corrected estimates), 75% of residents were women, and the mean (SD) age was 83.4 (7.3) years. Forty-three percent had no formal education and 40% completed the primary level. Health was declared as very good, good, fair, poor, and very poor by 15, 40, 30, 10, and 5% of residents, respectively. The mean (SD) number of chronic conditions was 2.9 (2.0), and 51% were independent or mild dependent in basic activities of daily living. In addition, 41% had cognitive impairment, 31% showed depressive symptoms, 12.8 and 12.9% had moderate/severe vision and hearing impairments, respectively, and for 43% internal contacts were scarce or none.

Table 1 displays results from the main model. Self-rated health was similar by sex and age group. Residents in private facilities and those who completed primary education had better health perception (adjusted odds ratios 0.57 and 0.64, respectively). The adjusted odds for worse health perception increased 18% for each additional chronic condition. Residents with any degree of dependency had between 1.81 and 2.37 times higher odds of worse perception than those functionally independent. Results further adjusted for cognitive status and depressive symptoms remained virtually unchanged (not shown).

**Table 1:** Factors associated with worse self-rated health in institutionalized older persons.

Factor	OR* (95% CI)	
	
	Unadjusted	Adjusted†
**Sex**		
Men	1	1
Women	1.00	1.02 (0.73 – 1.44)
**Age (years)**		
65 – 84	1	1
≥85	1.00	0.87 (0.63 – 1.20)
**Education**		
None	1	1
Primary	0.62	0.64 (0.43 – 0.95)
Secondary or more	0.51	0.66 (0.40 – 1.10)
**Facility**		
Public/concerted	1	1
Private	0.55	0.57 (0.37 – 0.87)
**Chronic conditions**		
1 unit increase	1.21	1.18 (1.07 – 1.28)
**Functional dependency**		
Independent	1	1
Mild	1.74	1.94 (1.11 – 3.37)
Moderate	2.00	2.37 (1.38 – 4.06)
Severe	1.74	1.81 (0.96 – 3.41)

* Odds ratio (95% confidence interval) of worse health perception for each category compared to the corresponding reference group (men, 65 – 84 years, no education, public facility, and functionally independent), except for chronic conditions, showing the odds ratio for an increase of one chronic condition.

† Adjusted for the remaining variables in the table.

We also assessed the independent associations of mental and social variables with self-rated health (Table 2). Cognitive status showed no association. The odds ratios for worse health perception were 4.04 and 10.45 for residents with mild and moderate/severe depressive symptoms compared to those without depressive symptoms, respectively. We also found an association with vision problems (odds ratio 2.05), but not with hearing impairment. In the social support realm, only internal contacts showed an independent association (odds ratio 1.42).

**Table 2:** Mental and social factors associated with worse self-rated health in institutionalized older persons.

Factor	Subjects	OR* (95% CI)
**Cognitive status**	484	
Normal		1
Impaired		1.01 (0.60 – 1.72)
**Depressive symptoms**	588	
Normal		1
Mild		4.04 (2.50 – 6.60)
Moderate/severe		10.45 (5.84 – 18.68)
**Vision**	556	
Good/mild		1
Moderate/severe		2.05 (1.36 – 3.08)
**Audition**	542	
Good/mild		1
Moderate/severe		1.21 (0.74 – 1.98)
**Internal contacts**	619	
Frequent/normal		1
Rare/nothing		1.42 (1.03 – 1.97)
**External contacts**	617	
Daily/weekly/monthly		1
Lower than monthly/nothing		1.08 (0.82 – 1.42)
**Relatives in facility**	622	
Yes		1
No		0.73 (0.48 – 1.12)

* Odds ratio (95% confidence interval) of worse health perception for each category compared to the corresponding reference group (normal cognition, no depressive symptoms, good/mild vision and audition, and present of internal, external contacts, and relatives in facility), adjusted for sex, age, education, type of facility, number of chronic conditions, and functional dependency.

Table 3 shows results stratified by sex, type of facility, and cognitive function. The pattern of determinants was similar between men and women, except for education and functional dependency. The better health perception in those with primary education was mainly attributed to women (*p *value for interaction 0.005), whereas the worse perception in functionally dependent residents was stronger for men (*p *value for interaction 0.082). We found no significant departures from the main effects by type of facility, although a qualitative modification of the sex effect was noted: in public facilities women rated their health worse than men, and the opposite came about in private nursing homes (*p *value for interaction 0.179). We finally explored the profile in each subgroup of cognitive status. In those with normal function effects were similar to those previously reported, but somewhat erratic patterns were observed in cognitively impaired residents. In particular, the effect of functional dependency changed its direction, with functionally dependent subjects showing better health perception than those independent (*p *value for interaction 0.315). Nevertheless, the reduced number of cognitively impaired individuals resulted in imprecise interval estimates in this subgroup and low statistical power of interaction tests.

**Table 3:** Factors associated with worse self-rated health by sex, type of facility, and cognition in institutionalized older persons.

Factor	OR* (95% CI)					
	
	Sex		Facility		Cognitive function	
			
	Men (*n *= 298)	Women (*n *= 332)	Public/concerted (*n *= 425)	Private (*n *= 205)	Normal (*n *= 308)	Impaired (*n *= 176)
**Sex**						
Men			1	1	1	1
Women			1.24 (0.87 – 1.77)	0.72 (0.38 – 1.36)	1.17 (0.74 – 1.86)	0.96 (0.53 – 1.76)
**Age (years)**						
65 – 84	1	1	1	1	1	1
≥85	0.70 (0.42 – 1.15)	0.92 (0.62 – 1.35)	0.89 (0.59 – 1.33)	0.75 (0.43 – 1.30)	0.59 (0.37 – 0.93)	1.13 (0.61 – 2.10)
**Education**						
None	1	1	1	1	1	1
Primary	1.17 (0.64 – 2.13)	0.53 (0.34 – 0.82)	0.58 (0.35 – 0.97)	0.71 (0.37 – 1.37)	0.82 (0.47 – 1.42)	0.52 (0.24 – 1.13)
Secondary	0.51 (0.22 – 1.17)	0.75 (0.38 – 1.51)	0.76 (0.40 – 1.44)	0.63 (0.29 – 1.34)	0.86 (0.48 – 1.53)	0.36 (0.12 – 1.11)
**Facility**						
Public/concerted	1	1			1	1
Private	0.68 (0.33 – 1.40)	0.51 (0.30 – 0.87)			0.54 (0.32 – 0.90)	0.58 (0.25 – 1.32)
**Chronic conditions**						
1 unit increase	1.25 (1.07 – 1.46)	1.16 (1.06 – 1.27)	1.23 (1.10 – 1.37)	1.09 (0.93 – 1.28)	1.20 (1.04 – 1.39)	1.12 (0.95 – 1.31)
**Functional dependency**						
Independent	1	1	1	1	1	1
Mild	3.09 (1.46 – 6.47)	1.61 (0.81 – 3.22)	2.29 (1.14 – 4.62)	1.37 (0.36 – 1.25)	1.75 (0.87 – 3.51)	0.49 (0.09 – 2.68)
Moderate	2.59 (1.31 – 5.09)	2.29 (1.14 – 4.62)	2.10 (1.19 – 3.73)	2.55 (1.71 – 9.15)	2.21 (1.17 – 4.18)	0.56 (0.10 – 3.12)
Severe	2.79 (1.16 – 6.70)	1.55 (0.74 – 3.22)	1.88 (0.76 – 4.58)	1.74 (0.49 – 6.25)	1.51 (0.60 – 3.81)	0.57 (0.10 – 3.24)

* Odds ratio (95% confidence interval) of worse health perception adjusted for sex, age, education, type of facility, number of chronic conditions, and functional dependency.

## Discussion

To our knowledge, this is the first study that examines factors associated with self-rated health in older people living in institutions. Apart from a strong effect of depressive symptoms, the main determinants of self-rated health in our institutionalized population were the number of illnesses and functional dependency. We also found an independent association with socioeconomic variables, such as type of facility and education, particularly among women.

In general terms, and compared to other institutionalized populations [17], this population can be described as being in relatively good level of health, functioning, and cognition. The facilities in our area are assisted living, skilled nursing, and mixed. Overall, 55% residents had no assigned caregiver, thus indicating a low need of care, assimilated to residential care.

A comparison of self-rated health in elders participating in our study with community-dwelling elders in a study conducted with similar methods in the same geographic area showed higher health perception ratings among institutionalized participants (55% versus 49% with very good and good self-rated health) [18]. This unexpected finding may be partly explained by general health status and functioning of these populations. In our facilities there is a large portion of residents with low needs for care, while there are also persons in the community that should be better in institutions, but remain in their homes. In addition, self-rated health, as a subjective matter, implies some comparative assessments [19] making context a relevant issue. When rating their health, individuals may make some "adjustment" by comparing their situation with that of others around. This phenomenon may lead to overly positive ratings in institutionalized populations, thus limiting its value. On the other hand, we think that some people unconsciously incorporate certain degree of complaint to their answers. We hypothesize that this group is proportionately higher in the community.

We did not find overall associations of self-rated health with demographic variables, but some subgroup results are worth mentioning. In the community [18], we previously reported a clear better health perception in the oldest group (≥ 85 years) as compared with younger individuals (65–74 years), a behaviour consistent with other reports [20]. In the present study we found a similar effect only in the subgroup of residents with normal cognition. Regarding sex, we appreciated some differences between men and women concerning the effects of education and type of facility, with a better health perception more clearly associated with higher educational levels and private facilities among women than men. Though these effect modifications were weak, it seems that sex and socioeconomic status may interact in determining health perception.

Cognitive function is an important variable that could play a role in institutionalized populations. Although some degree of misclassification is likely, we found no relevant effect of cognitive status on health perception. In addition, the pattern of determinants remained very similar after adjusting for cognitive status and also after excluding cognitively impaired residents. On the other hand, we found clear inconsistencies in those cognitively impaired, but random errors derived from the small sample size of this subgroup impede a proper appraisal. Some work suggests that cognitively impaired individuals can provide equivalent assessment of their health status [21,22], but in-depth studies on the role of cognitive function as determinant of self-rated health could add valuable knowledge.

Depression was highly prevalent and showed a very strong, independent effect on self-rated health. Similar results were observed in our population of community-dwelling older people [18], as well as in other communities [23]. Accordingly, this great dependence on depressive symptoms must always be considered when interpreting health ratings.

The strengths of this study include the use of a probabilistic sample, the high response rate, and the analysis of a wide panel of relevant variables. However, some limitations are worth mentioning. First, the SPMSQ and MDS-COGS cognition scales used in this study, as well as the Geriatric Depression Scale, have not been validated for Spanish population, but the translation of most questions is straightforward and no important misclassification biases are expected when applied to our setting. Second, an important part of the distinctive value of studies conducted in nursing homes resides on cognitively impaired residents and our work achieved low power in this subgroup.

## Conclusion

In summary, we found that some consistent determinants of health perception in the community, such as chronic conditions and functional dependency, are also relevant in institutions, but new features emerge in this setting, particularly the very strong effect of depressive symptoms and the important role of type of facility and educational level. It remains important to elucidate issues in cognitively impaired persons through an adequately powered study. Finally, it should be pointed out that our population, with a favourable health and function profile, can differ from other nursing home populations, making highly constructive the research on health perceptions in various institutional settings to provide sensible contrasts.

## Competing interests

The author(s) declare that they have no competing interests.

## Authors' contributions

All authors contributed to concept and design, analysis and interpretation of data, preparation of manuscript, and have given final approval for publication. EV and JD also contributed to the fieldwork and acquisition of data.

## Pre-publication history

The pre-publication history for this paper can be accessed here:


